# Pulmonary and Liver Toxocariasis Mimicking Metastatic Tumors in a Patient with Colon Cancer

**DOI:** 10.3390/diagnostics14010058

**Published:** 2023-12-26

**Authors:** Miju Cheon, Jang Yoo

**Affiliations:** Department of Nuclear Medicine, Veterans Health Service Medical Center, Seoul 05368, Republic of Korea

**Keywords:** ^18^F-FDG, PET, CT, toxocariasis

## Abstract

Toxocariasis is an uncommon cause of multiple cavitary lung lesions and an ill-defined liver lesion. We herein report a patient with lung and liver toxocariasis, which mimicked metastatic lesions of colon cancer on ^18^F-FDG PET–CT and chest and abdominal CT performed for cancer staging after diagnosis of colon cancer. The patient was diagnosed with lung and liver toxocariasis by a positive enzyme-linked immunosorbent assay. Lung toxocariasis may occur as multiple cavitary lung lesions, and liver toxocariasis may appear as a solitary ill-defined nodule, which may be misdiagnosed as metastatic tumors. Clinicians should consider toxocariasis when multiple cavitary lung lesions and a solitary ill-defined focal liver lesion are detected, especially in a patient with cancer.

A 76-year-old man was diagnosed with colon cancer via a routine health examination, including a colonoscopy. As for colon cancer staging, chest computed tomography (CT), abdominal CT, and ^18^F-FDG positron emission tomography–CT (PET–CT) were performed. ^18^F-FDG PET–CT revealed a hypermetabolic mass in the sigmoid colon, representing colon cancer. It also demonstrated a mild hypermetabolic nodule in segment 5/6 of the liver and multiple mild hypermetabolic cavitary nodules in both lungs (Figure 1). Based on these findings, we suspected sigmoid carcinoma metastasized to the lung and liver as the first differential diagnosis.

Chest contrast-enhanced CT revealed multiple peribronchial, centrilobular, and cavitary nodules in both lungs (Figure 2), and they have upper lung predominance. The radiologist considered this to be pulmonary metastasis from colorectal cancer first and an infectious condition second. Abdominal CT showed a 2 cm sized, ill-defined lesion in segment 5/6 of the liver (Figure 3). As a differential diagnosis for the solitary hepatic lesion, we considered sclerosing hemangioma, inflammatory pseudotumor, or metastasis. Laboratory results showed only a mild increase in the eosinophil ratio (6.6%). Except for that, no abnormalities were found in the coagulation system or general biochemical tests. Serologic markers for hepatitis B and C, acid-fast bacteria (AFB) stain, and AFP culture were negative. Regarding tumor markers, carcinoembryonic antigen and carbohydrate antigen 19-9 were normal. At that time, he did not take any medication or show signs of infection or other systemic symptoms such as fever or night sweats. In addition, his exact history of previously eating uncooked cow liver or meat is unclear.

Liver magnetic resonance imaging (MRI) was performed to differentiate the pulmonary and hepatic lesions from another possible diagnosis. It revealed a 1.5 cm ill-defined nodular lesion contiguously extending into the liver capsule (Figure 4). These findings mostly likely represented liver fluke disease. The diagnosis of toxocariasis was confirmed through the presence of antibodies to *Toxocara canis* by enzyme-linked immunoassay (ELISA). Other parasite antibody tests, including *Clonorchiasis sinesis*, *Paragonimus westermani*, *Taenia solium*, *Spirometra mansoni*, *Fasciola hepatica*, *Amoeba*, *Trichinella spiralis*, and *Schistosoma*, all yielded negative results. Eventually, his colon cancer was treated with laparoscopic low anterior resection. After discharge, the patient took oral albendazole by himself. After 3 months, chest and abdominal CT was performed for the postoperative routine follow-up, and it showed a shrinkage of all pulmonary and hepatic lesions compared to the previous imaging. Finally, after 5 months, the entire multiple cavitary lung and the hepatic nodules disappeared (Figure 2 and Figure 3). In addition, the eosinophil ratio decreased from 6.6% to 0.4%. 

Human toxocariasis is a neglected soil-transmitted helminth infection from dogs and cats caused by larvae of *Toxocaria canis* or *Toxocara cati*, respectively. It causes various diseases, from compartmentalized to generalized visceral larva migrans [1]. Toxocariasis is a more common parasitic disease in Africa and South East Asia than in Western countries. In particular, in Korea, the prevalence of toxocariasis has increased among Korean adults due to the habit of eating uncooked cow liver or raw fish [2]. Several reports deal with imaging findings of pulmonary or hepatic toxocariasis [3,4,5,6]. Lung involvement of toxocariasis rarely causes multiple nodules [7,8]. Lee et al. stated the pulmonary lesions of toxocariasis tend to be multiple lesions with lower lung predominance and ill-defined ground-glass opacitiess with or without solid portions [9]. Hepatic toxocariasis usually appears as multiple ill-defined, oval-shaped, and low-attenuating nodules on CT. On CT or MRI imaging, hepatic lesions are seen as multiple ill-defined, oval lesions that measure 1.0–1.5 cm in diameter [10]. Sometimes, the lesion may be angular or trapezoid. The lesions are usually best seen on the portal venous phase in dynamic contrast-enhanced CT and MRI imaging; the lesions are either not seen or only faintly seen on arterial and equilibrium phases. However, in this case, the imaging was performed for cancer staging, the patient had no symptoms, eosinophilia was not high enough to suspect parasitic infection, and the imaging findings were not typical to suggest parasitic infection. In particular, the lung and liver are common sites of colorectal cancer metastasis. Hence, the possibility of metastatic cancer had to be considered first. Due to toxocariasis’s non-specific appearance on CT, they can often be mistaken for other conditions. Also, ^18^F-FDG PET–CT is currently a valuable diagnostic tool for cancer staging; FDG-avid inflammatory lesions can be misinterpreted as metastatic lesions. To our knowledge, there has been no case where toxocariasis was accidentally discovered during the diagnosis of any cancer, and in particular, there were no reports of simultaneous involvement of the lung and liver. Based on this interesting case, we suggest that clinicians should consider toxocariasis when patients with cancer present with solitary hepatic or multiple cavitary pulmonary nodules associated with eosinophilia and should perform serologic tests to exclude toxocariasis.

## Figures and Tables

**Figure 1:**
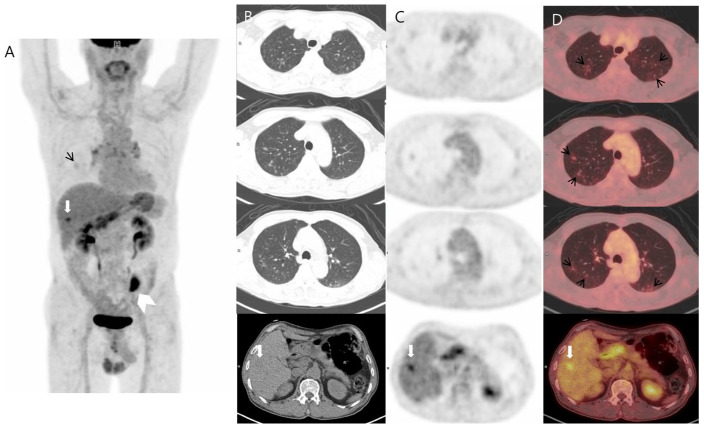
Three-dimensional maximum intensity projection (**A**) and transaxial images ((**B**) CT; (**C**) PET; (**D**) fusion) of ^18^F-FDG PET–CT demonstrated, in addition to a hypermetabolic mass in the sigmoid colon (white arrowhead), abnormal multiple cavitary pulmonary nodules with mildly increased FDG uptake (black short arrows) and a focal hypermetabolic nodule in the right hepatic lobe (white arrow). The maximum standard uptake values were 1.81 for the pulmonary nodule and 4.8 for the liver lesion.

**Figure 2:**
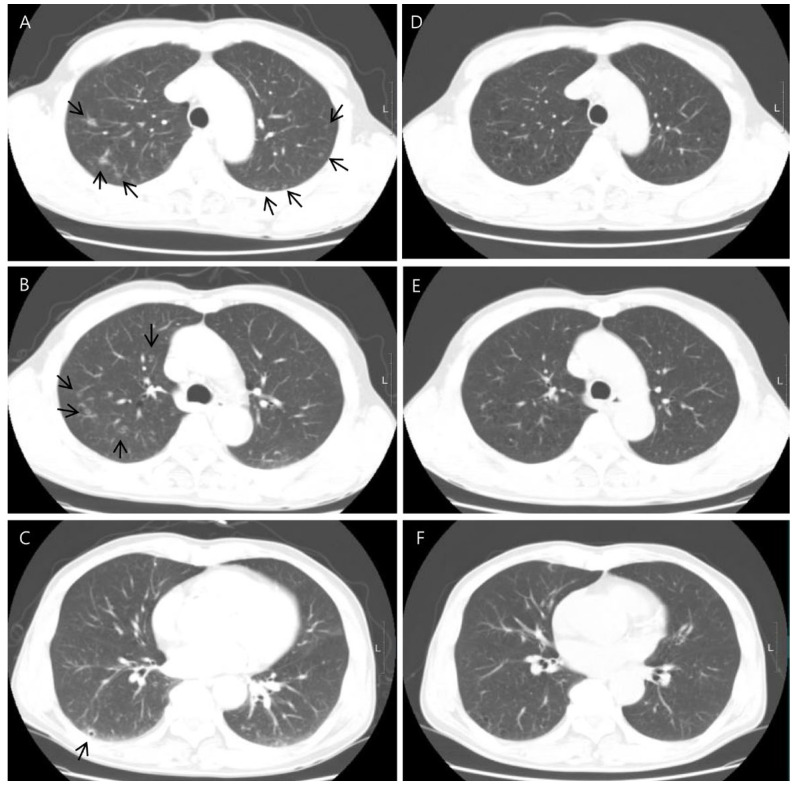
Axial chest CT showed multiple cavitary lung nodules in both lungs with upper lung zone predominance ((**A**–**C**), arrows). CT images obtained 5 months after showed the disappearance of the multiple cavitary nodules (**D**–**F**).

**Figure 3:**
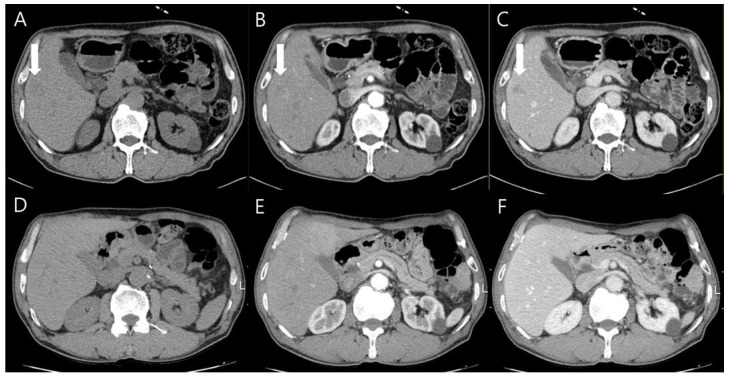
Abdominal CT images showed the solitary 2 cm ill-defined nodule in segment 5/6 of the liver ((**A**–**C**), arrows). It was presented as an ill-defined low-attenuation nodule in the pre-contrast phase (**A**), poorly delineated in the arterial phase (**B**), and also visible in the portal phase (**C**). After 6 months, the nodule was invisible in all phases (**D**–**F**).

**Figure 4:**
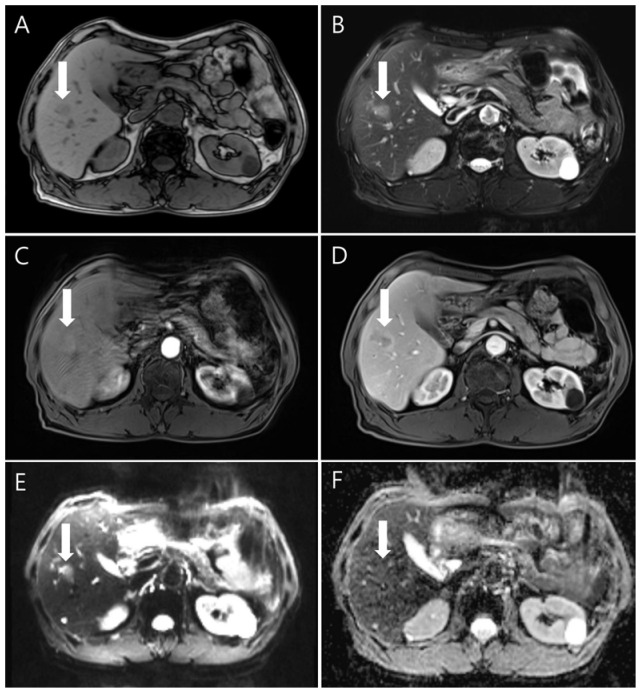
The gadolinium-enhanced dynamic MRI findings showed the nodule (white arrows) with low signal intensity on the unenhanced T1-weighted image (**A**) and high signal intensity on an unenhanced T2-weighted image (**B**). In dynamic imaging, the lesion showed slightly high signal intensity in the arterial phase (**C**) and rim enhancement in the portal venous phase (**D**). Diffusion-weighted imaging (**E**) showed indistinct high signal intensity; the apparent diffusion coefficient mapping imaging (**F**) was isointense.

## Data Availability

The data that support the findings of this study are available from the corresponding author M.C. upon reasonable request.
